# Repurposing Drugs in Oncology (ReDO)—chloroquine and hydroxychloroquine as anti-cancer agents

**DOI:** 10.3332/ecancer.2017.781

**Published:** 2017-11-23

**Authors:** Ciska Verbaanderd, Hannelore Maes, Marco B Schaaf, Vikas P Sukhatme, Pan Pantziarka, Vidula Sukhatme, Patrizia Agostinis, Gauthier Bouche

**Affiliations:** 1 Anticancer Fund, Brussels, 1853 Strombeek-Bever, Belgium; 2 Cell Death Research and Therapy Lab, Department of Cellular and Molecular Medicine, KU Leuven, 3000 Leuven, Belgium; 3 Clinical Pharmacology and Pharmacotherapy, Department of Pharmaceutical and Pharmacological Sciences, KU Leuven, 3000 Leuven, Belgium; 4 GlobalCures, Inc, Newton, MA 02459, USA; 5 Beth Israel Deaconess Medical Center and Harvard Medical School, Boston, MA 02215, USA; Current address: Emory School of Medicine, Atlanta, GA 30322, USA; 6 The George Pantziarka TP53 Trust, London KT1 2JP, UK

**Keywords:** Repurposing Drugs in Oncology (ReDO) project, drug repositioning, chloroquine (CQ), hydroxychloroquine (HCQ), neoplasms, antineoplastic agents, anti-malarial agents

## Abstract

Chloroquine (CQ) and hydroxychloroquine (HCQ) are well-known 4-aminoquinoline antimalarial agents. Scientific evidence also supports the use of CQ and HCQ in the treatment of cancer. Overall, preclinical studies support CQ and HCQ use in anti-cancer therapy, especially in combination with conventional anti-cancer treatments since they are able to sensitise tumour cells to a variety of drugs, potentiating the therapeutic activity. Thus far, clinical results are mostly in favour of the repurposing of CQ. However, over 30 clinical studies are still evaluating the activity of both CQ and HCQ in different cancer types and in combination with various standard treatments. Interestingly, CQ and HCQ exert effects both on cancer cells and on the tumour microenvironment. In addition to inhibition of the autophagic flux, which is the most studied anti-cancer effect of CQ and HCQ, these drugs affect the Toll-like receptor 9, p53 and CXCR4-CXCL12 pathway in cancer cells. In the tumour stroma, CQ was shown to affect the tumour vasculature, cancer-associated fibroblasts and the immune system. The evidence reviewed in this paper indicates that both CQ and HCQ deserve further clinical investigations in several cancer types. Special attention about the drug (CQ versus HCQ), the dose and the schedule of administration should be taken in the design of new trials.

## Introduction

Chloroquine (CQ) and hydroxychloroquine (HCQ) are both 4-aminoquinoline agents that have been used for more than 70 and 50 years, respectively, to prevent or to treat malarial infections and later also for treating discoid and systemic lupus erythematosus and rheumatoid arthritis. Although HCQ and CQ differ only by one hydroxyl group, the addition of this hydroxyl group results in an important decrease in toxicity, while the efficacy remains constant, at least for malaria [1]. Both drugs are available as generic products and mentioned on the WHO list of essential medicines. Frequently used trade names of CQ include Avloclor, Nivaquine or Aralen, and the most frequently used trade name for HCQ is Plaquenil.

The mechanisms of action of CQ and HCQ against the malarial *Plasmodium* parasite and against the auto-immune disorders for which they are approved are well known [2–6].

## Dosage

The dosage of CQ depends on the indication [3, 4]. It should be noted that CQ is often marketed as chloroquine phosphate (CQ-phosphate) in tablets of 250 mg, which corresponds to about 150 mg of CQ. All doses mentioned below are doses of CQ-phosphate. High doses (1 g of CQ-phosphate per day) are administered in acute phases of malaria or amoebic hepatitis, but only for one or two days. The usual dose for long-term use (rheumatoid arthritis and lupus) is 250 mg of CQ-phosphate per day. For HCQ, doses for long-term use range between 200 and 400 mg per day. Long-term administration of CQ and HCQ in children is not recommended, but doses for long-term treatment between 2 and 5 mg/kg for HCQ have been reported [7].

## Toxicity

Short-term administration of CQ or HCQ rarely causes severe side effects. Longer exposure has been associated with some serious though uncommon adverse events [3], including cardiomyopathy [8], irreversible retinal toxicity [9, 10], bone marrow suppression [11] and hypoglycaemia [12]. The risk of retinopathy is increased with large cumulative doses of HCQ (>1000 g). However, daily doses up to 400 mg of HCQ or 250 mg CQ for several years are considered to carry an acceptable risk for CQ-induced retinopathies, with the exception of individuals of short stature [13]. It is advised that patients receiving chronic CQ or HCQ therapy be monitored through regular ophthalmic examinations (3–6 month intervals), full blood counts and blood glucose level checks. CQ has been associated with some cases of diffuse parenchymal lung disease and drug rash with eosinophilia and systemic symptoms (DRESS) syndrome [3]. In case of long-term HCQ exposure, skeletal muscle function and tendon reflexes should be monitored for weakness.

For both CQ and HCQ, specific caution is advised in patients suffering from impaired hepatic function (especially when associated with cirrhosis), porphyria, renal disease, epilepsy, psoriasis, glucose-6-phosphate dehydrogenase deficiency and known hypersensitivity to 4-aminoquinoline compounds [3].

## Bioavailability

CQ and HCQ are amphiphilic weak bases with the ability to cross cell membranes easily, which is important for their mechanism of action in malaria treatment and prophylaxis. CQ and HCQ are partially protonated at the physiologic pH (7.4), but they can be trapped in lysosomes (pH 4–5) because of bi-protonation [14]. CQ has pKa values of 8.4 and 10.2, while HCQ has pKa values of 8.3 and 9.7 [14, 15].

Both CQ and HCQ have a high bioavailability, 89% and 74% respectively, and a large distribution volume after oral administration. Roughly, 50%–70% of CQ is protein-bound in the plasma [3]. The terminal elimination half-life of CQ is 1–2 months and for HCQ approximately 50 days in blood (32 days in plasma). Both drugs are partially metabolised by hepatic dealkylation, but they differ in the number of the metabolites produced. The active metabolites of CQ are monodesethylchloroquine and bisdesethylchloroquine, while HCQ has one extra active metabolite, namely desethylhydroxychloroquine. Moreover, CQ and HCQ are slowly excreted and may still be detected in urine several months after administration [3, 4, 9].

## Pre-clinical evidence in cancer—*in vivo*

CQ and HCQ have been extensively studied both *in vitro* and *in vivo* in various cancer types. This paper focuses on the results from *in vivo* research, since this is most relevant to clinical practice. Both drugs can be administered as monotherapy or as adjuvant agents to increase the efficacy and to limit drug resistance of standard anti-cancer therapy.

### 

#### Monotherapy

Table 1 lists the main characteristics [animal models, tumour types, animal (H)CQ doses and human equivalent doses (HED)] of the *in vivo* studies performed with CQ or HCQ alone.

Starting with *in vivo* studies that observed beneficial effects of CQ administration in cancer, Jutten *et al* noted a delayed tumour growth in mice bearing epidermal growth factor receptor (EGFR)-overexpressing glioblastoma xenografts in response to CQ administration. In addition, the time to reach four times the initial tumour volume was significantly longer in the CQ-treated group [16]. Kim *et al* confirmed this observation in another glioblastoma xenograft mouse study, where CQ was injected intracranially. They observed that the number of mitotic cells was significantly reduced and the number of apoptotic cells was increased after CQ administration [17]. In addition, a significant reduction of tumour volume and tumour incidence was shown by Song *et al* [18] in mice bearing liver cancer stem cells and Hu *et al* [19] observed significant tumour growth and weight reduction in an orthotopic xenograft model of liver cancer after CQ administration. Lakhter *et al* [20] demonstrated that CQ significantly reduced both tumour volume and tumour mass in a human melanoma xenograft model. Zheng *et al* [21] showed reduced tumour progression and prolonged survival time (not significant) in colon cancer-bearing mice when administering either 25 or 50 mg/kg of CQ.

Doses of 25 and 50 mg/kg of CQ both significantly increased survival time and reduced primary tumour volume in mice implanted with a highly metastasizing breast cancer cell line, as shown by Jiang *et al*. Interestingly, the number and diameter of lung metastases was reduced as well, and CQ enhanced tumour cell apoptosis in the high dose group [22].

The incidence of mammary tumours and their growth rate was significantly lower and tumour onset was delayed in CQ-pre-treated rats after being subjected to mammary adenocarcinoma induction using N-methyl-N-nitrosourea (NMU), as shown by Loehberg *et al*. In wild-type BALB/c mice transplanted with mammary ducts of BALB/c p53-null mice, CQ pre-treatment did not affect tumour incidence [23]. Maclean *et al* confirmed that CQ could not prevent spontaneous tumour formation in p53-deficient mice. In contrast, intermittent CQ administration significantly reduced the tumour development and doubled the overall survival (OS) of E*μ*-Myc mice [24].

Furthermore, Sun *et al* showed that CQ administration is effective in reducing tumour growth in rats with established hepatocarcinoma. In contrast, CQ promoted tumour development in the earlier so-called dysplastic stage, clearly illustrating the dual role of autophagy in tumour formation (see section on mechanisms of action) [25]*.* Finally, Maes *et al* [26] reported that either a dose of 50 mg/kg or a dose of 100 mg/kg of CQ can reduce tumour growth and cell proliferation*,* dependent on the cell type*.* Of note, this study showed that CQ not only inhibits autophagy but also affects the tumour microenvironment and tumour vasculature. The exact working mechanisms will be clarified in the section on mechanisms of action.

Some studies noted that the efficacy of CQ application in anti-cancer therapy depends on the tumour type that is being treated and suggested that the autophagy dependency of tumour cells might play a role [27, 28]. For example, tumour growth was significantly reduced in an MDAMB231 xenograft mouse model, but not in an MCF-7 xenograft mouse model, while both models showed signs of autophagy inhibition after CQ treatment [28]*.* A similar observation can be made when comparing CQ efficacy in pancreatic cancer mouse models and a lung cancer mouse model: CQ significantly slowed down tumour growth and increased survival in the first, but not in the latter [27]. Another study by Hiraki *et al* [29] investigated the effects of CQ in various *in vivo* cancer models and demonstrated that CQ is more effective in connective tissue-rich Bashford and Brown–Pearce tumours than in Ehrlich, Yoshida and MH134 tumours.

A lack of efficacy in certain tumour models could potentially be explained by a study performed by Pellegrini *et al* exploring the effects of CQ under acidic conditions, which mimics the tumour environment. CQ bi-protonation under those conditions could impede cytotoxicity, because the cellular uptake of CQ is reduced. This observation highlights a possible limitation of CQ in anti-cancer therapy. However, the sensitivity of tumour cells might be restored using tumour pH-modulating agents [30]*.* Ironically, hypoxic cells that can increase acidification of the extracellular space through anaerobe glycolysis are often more autophagy-dependent and, therefore, more sensitive to CQ treatment, as shown by *in vitro* studies [31]*.*

A limited amount of studies reported potential detrimental effects of CQ and HCQ in *in vivo* cancer models. First, CQ pre-treatment of rats one week before a subcutaneous injection with mammary adenocarcinoma and follow-up treatment for 18 days following this event significantly enhanced tumour weight and volume in these rats [32]. Second, in a 1966 paper, very low CQ doses (0.2 mg/2 days) led to a more infiltrative morphological pattern of the advancing margin of subcutaneously transplanted mammary carcinoma in mice [33]. Third, malignant tumour growth and metastasis of Ras(V12) cells is observed in transgenic drosophila models after CQ administration [34]*.* Importantly, HCQ promoted tumour growth in Ras-driven pancreatic tumours developing without p53(Kras^G12D/+^p53^–/–^) [35]. Collectively, these results on a possible detrimental effect of (H)CQ emphasise the importance of the specific tumour setting and tumour characteristics when targeting autophagy (see section on mechanisms of action) [35, 36]*.*

#### Combination therapy

Table 2 summarises the information from articles that studied the effect of CQ (*n* = 46) or HCQ (*n* = 5) *in vivo* in combination with other therapies. A more interesting and still under-explored treatment approach for a complex disease such as cancer is to combine various anti-cancer agents acting at different levels in the tumour cells and microenvironment [37]. Interestingly, CQ and HCQ have already been tested in combination with over 40 other drugs in preclinical cancer research. Both CQ and HCQ can effectively increase the efficacy of various anti-cancer drugs, which is further explained in the section on mechanisms of action. Therapies used in combination with CQ or HCQ include chemotherapeutic drugs, tyrosine kinase inhibitors, various monoclonal antibodies, hormone therapies and radiotherapy (Table 2).

## Human data

Numerous clinical trials in which either CQ or HCQ is being used to treat patients with a range of cancer types are registered in clinical trial databases. In clinical trials, these drugs are most often administered in combination with other anti-cancer agents. More information on the registered clinical trials is provided in Tables 3 and 4, for CQ and HCQ, respectively. Few trials have been completed. Therefore, limited published data are available on the safety and therapeutic efficacy of these antimalarial drugs in cancer. A schematic overview of the published clinical trial data of CQ and HCQ can be found in Tables 5 and 6, respectively.

In the next section, the clinical effects of CQ and HCQ will be discussed separately because important differences can be observed in toxicity and efficacy of both drugs.

### CQ

#### Glioma and brain metastases

In May 1998, one of the first clinical trials on CQ use in cancer was started, which was an open, prospective, randomised controlled study with 18 glioblastoma multiforme (GBM) patients [38]. The test group consisted of nine patients who received 150 mg CQ daily after resection of the lesion, in addition to radiotherapy (total dose of 6000 Gy) and four cycles of carmustine-chemotherapy every six weeks (200 mg/m^2^), while the nine patients in the control group received placebo instead of CQ. In the abstract of this study, the authors reported that adjuvant CQ administration significantly enhanced patient survival [33 ± 5 months for CQ-treated patients and 11 ± 2 months for controls (p < 0.0002)]. Due to some inconsistencies in the report, the calculation of the mean survival in the CQ-treated group is unclear, but the Kaplan Meier analysis remains significant. A higher seizure frequency was observed in the CQ-treated group and could not be explained. However, standard antiepileptic treatment was reported to easily suppress these seizures. The same group of researchers started a similar randomised, double blind, placebo-controlled study in October 2000 [39]. In this second study, 15 GBM patients received 150 mg CQ each day for 12 months after surgery in combination with their conventional anti-cancer therapy, four cycles of carmustine-chemotherapy every five weeks (200 mg/m^2^) and a total radiation dose of 60 Gy; the other 15 patients received adjuvant placebo treatment. A median survival time of 24 months was observed in the CQ-treated group, as compared with 11 months in the control group. In addition, the hazard ratio for death was approximately half as large in the patients receiving CQ though this was not statistically significant (hazard ratio: 0.52, [95% CI 0.21–1.26, *p* = 0.139]). No important adverse effects were noted in this trial. The small sample size is an important limitation in both studies, and larger clinical trials are needed to confirm the efficacy of CQ in GBM therapy [39, 40]. In a retrospective study, the same research group looked at data collected over five years from 41 GBM patients in Mexico who received adjuvant CQ therapy and did not participate in the previously mentioned clinical trials [41]. The mean survival time of these CQ-treated patients was significantly longer compared with a control group of 82 glioblastoma patients [25 ± 3.4 months and 11.4 ± 1.3 months after surgery respectively (*p* = 0.000)].

After the observation of promising outcomes in five recurrent GBM patients treated with 250 mg CQ a day and reirradiation for 20 months [42], a phase-2 clinical trial tested the effects of CQ as a radio-sensitising agent in patients with brain metastases [43]. In this trial, 39 patients were administered whole-brain irradiation (30 Gy in 10 fractions over two weeks) in combination with a daily dose of 150 mg CQ for four weeks, while 34 patients received placebo instead of CQ in addition to the same radiation treatment. The overall response rate or OS did not improve after CQ administration. However, the progression-free survival of brain metastases rate was increased (CQ-treated group: 83.9% [95% CI 69.4–98.4] and control group: 55.1% [95% CI 33.6–77.6] (at one year), relative risk: 0.31 [95% CI 0.1–0.9, *p* = 0.046]). The absence of adverse effects and the improved local control of brain metastases indicate that CQ might be a useful addition to whole brain irradiation in patients with brain metastases. In a prospective, single-cohort study of 20 patients with brain metastases from solid tumours, 250 mg CQ daily was administered for five weeks in combination with whole-brain irradiation [44]. The intracranial response rate corresponded to an objective clinical response of 93% after three months of whole-brain irradiation, there was a slight, positive trend in OS (median OS of 5.7 months, compared with 4.2 months for patients in class II estimated by the radiation therapy oncology group recursive partitioning analysis), and no adverse reactions were detected. Finally, two case reports mentioned unusual skin reactions after concomitant use of CQ and radiation, illustrating the radio-sensitising effect of CQ [45, 46].

In a paediatric patient with a recurrent BRAF V600E mutant brainstem ganglioglioma, tumour growth was blocked and vemurafenib sensitivity restored following treatment with 150 mg CQ daily for at least 30 months [47, 48]. The same research group reported *in vitro* and *ex vivo* data showing that autophagy inhibition was able to improve the response to BRAF inhibition in resistant tumour cells [48]. Next, CQ was administered to two patients with acquired resistance to BRAF inhibition. The first patient was treated with standard doses of vemurafenib plus 250 mg daily of CQ during focal radiation of large primary lesions. Vemurafenib was continued and the CQ dose was increased to 500 mg daily after completion of radiation. A rapid favourable clinical response to the combination therapy was observed in as little as six weeks and was maintained for seven months, at which point the patient had to stop therapy for unrelated medical issues. The second patient was treated with 500 mg CQ daily in combination with standard dosing of vemurafenib. Acquired resistance to vemurafenib was overcome within four weeks of the addition of CQ and clinical improvement could be observed, which was maintained for two and a half months. However, therapy had to be stopped and the family chose to pursue palliative therapy afterwards.

#### Multiple myeloma

Eleven patients with relapsed and refractory multiple myeloma were enrolled and treated with 500 mg CQ daily (on days 1–14 and 22–35) in addition to bortezomib and cyclophosphamide (administered orally twice daily) in a phase-2 clinical trial [49]. Of these eleven patients, only eight patients were evaluable. CQ was able to partially restore the bortezomib sensitivity: three patients had a partial response, one had stable disease and four had progression as best responses.

The adjuvant therapeutic effect of 250 mg CQ twice daily in combination with cyclophosphamide and prednisone was tested for a period of ten days in 38 myeloma patients [50]. Twenty patients received cyclophosphamide and prednisone, while the other 18 patients received extra treatment with CQ and caffeine, but no additional response was observed in the CQ-treated patient group.

### HCQ

#### Solid cancers

The effect of HCQ and temsirolimus combination therapy was tested in 27 patients with advanced solid cancer during a phase-1 dose-escalating study and subsequently in 13 patients with metastatic melanoma at the phase-2 recommended dose [51]. No patient experienced an objective response but 19 out of the 27 phase-1 patients (73%) and 9 out of 13 melanoma patients (69%) had stable disease. In patients with stable disease, HCQ addition was shown to produce metabolic stress in the tumours. Inhibition of autophagy (see section on mechanisms of action), measured by counting the number of autophagic vacuoles per cell in tumour tissues and peripheral blood mononuclear cells of patients, was only noted in patients receiving at least 1200 mg HCQ daily. This study recommends an adjuvant HCQ dose of 600 mg, twice daily.

Next, the combinatory effect of HCQ and temozolomide was investigated in 40 cancer patients with advanced solid tumours and melanoma, and the recommended dose of 600 mg twice daily was confirmed [52]. HCQ was shown to successfully inhibit autophagy, as evidenced by the significant accumulation of autophagic vacuoles in peripheral blood mononuclear cells (mean autophagic vacuole counts: 2.19 at baseline, 2.45 after HCQ treatment, 3.84 after treatment with HCQ plus TMZ [difference between HCQ plus TMZ and baseline: *p* = 0.0007, difference between HCQ plus TMZ and HCQ only: *p* = 0.0034]).

The safety and preliminary efficacy of HCQ and vorinostat combination treatment was tested during a phase-1 study in 27 patients with advanced solid tumours [53]. In this study, the maximum-tolerated HCQ dose was set at 600 mg daily in combination with 400-mg vorinostat. A confirmed durable partial response was observed in a renal cell carcinoma patient, and prolonged stable disease was seen in two colorectal cancer patients. In contrast to the previous study, autophagy was not significantly affected.

Finally, in a pilot retrospective study, 25 stage-IV cancer patients (various types) who had no clinical response to maximally tolerated chemotherapy and to first-line metronomic chemotherapy were treated with sirolimus (2 mg/day) and the autophagy inhibitor HCQ (400 mg/day) in addition to their current metronomic chemotherapy for at least three months. The therapy was reported to be relatively safe, and the overall response rate was 40%, with an 84% disease control rate [54]. However, this was a retrospective analysis requiring cautious interpretation.

#### Glioblastoma

The efficacy and safety of HCQ was studied in combination with radiotherapy and temozolomide in 92 GBM patients during a phase-1–2 study [55]. This study indicated a maximum tolerated dose (MTD) of 600 mg HCQ a day in this therapeutic setting. OS did not seem to be affected in comparison with the temozolomide arm of the trial reported by Stupp [56], and autophagy was not found to be consistently inhibited in all patients.

#### Lung cancer

The combination of HCQ with erlotinib can be used safely in daily doses of 150 mg erlotinib and 1000 mg HCQ, as determined by a phase-1 study in 27 patients with advanced non-small cell lung cancer (NSCLC) [57]. Of the 19 patients who remained in the study, one had a partial response and four had stable disease as best response. Subsequent ophthalmic surveillance on seven trial participants who had taken HCQ for a duration longer than six months showed that retinal toxicity occurred in two patients after 11 and 17 months of exposure [58].

This highlights the importance of retinal toxicity monitoring (via high-resolution spectral-domain optical coherence tomography, fundus auto fluorescence imaging, Humphrey visual field testing and multifocal electroretinography) during clinical trials with HCQ.

#### Multiple myeloma

During a phase-1 study, the safety of the combination of HCQ and bortezomib was explored in 25 patients with relapsed or refractory myeloma [59]. A dose of 600 mg HCQ twice daily was reported to be safe and tolerable in combination with standard doses of bortezomib. The increase in the number of autophagic vacuoles was not significantly associated with clinical response or HCQ exposure. Of 22 evaluable patients, three (14%) had very good partial responses, three (14%) had minor responses, ten (45%) had stable disease for at least one cycle and six (27%) had immediate progression.

#### Pancreatic cancer

The safety of the combination of pre-operative HCQ (1200 mg daily) and gemcitabine administration was demonstrated in 35 patients with pancreatic adenocarcinoma in a phase-1–2 trial [60]. This study reported promising clinical response markers (e.g. CA 19–9 biomarker and R0 resection rate). An exploratory analysis showed significantly improved disease-free survival and OS (15.03 versus 6.9 months and 34.83 versus 10.83 months, respectively) in patients for whom autophagy was sufficiently inhibited (*n* = 8) (at least 51% increase in the autophagy marker LC3B-II in peripheral blood mononuclear cells) compared with other patients (*n* = 9).

Next, a phase-2 study investigated the safety and efficacy of HCQ monotherapy with either 400 or 600 mg two times a day in 20 patients with previously treated metastatic pancreatic cancer, but no significant differences were observed between groups [61]. In addition, inhibition of autophagy could not be achieved consistently, as shown by LC3B-II analysis in the lymphocytes of patients, and the two-month progression-free survival rate was only 10%.

#### Sarcoma

The combination of 1 mg sirolimus and 200 mg HCQ twice daily for two weeks was tested in ten sarcoma patients who had failed first-line treatment [62]. This study started from the hypothesis that there is metabolic symbiotic relationship between cancer-associated fibroblasts (CAFs) and sarcoma cells (see mechanisms of action). The study showed that this relationship might be altered by treatment with sirolimus and HCQ as glycolysis was inhibited within the tumours. Based on FDG PET response criteria, two weeks after treatment initiation, six patients showed partial response, three had stable disease and one had progressive disease. However, most patients discontinued treatment before the initially planned eight-week response assessment, for disease progression.

## Mechanism of action

Multiple hypotheses have been proposed on how CQ and HCQ exert their anti-cancer activity. Most studies reported the direct action of these drugs on cancer cells, but more recent studies have also mentioned important effects of CQ and HCQ on the tumour microenvironment. Based on preclinical studies, it is safe to say that CQ and HCQ have multiple mechanisms of action that might complement each other.

The most relevant and evidence-based mechanisms of action of CQ and HCQ in anti-cancer treatment will be briefly explained in the next section. The benefits of combining these antimalarial drugs with existing anti-cancer treatments will also be described. In the final section, the variation in sensitivity of cancer patients to CQ and HCQ therapy will be clarified.

### Direct anti-tumour effects

The main and most studied anti-cancer effect of CQ and HCQ is the inhibition of autophagy, but other preclinically proven anti-cancer activities of the antimalarial agents include influencing the TLR9/nuclear factor kappa B (NF-κB) signalling pathway, the CXCL12/CXCR4 signalling pathway and the p53 pathway.

#### Autophagy inhibition

Autophagy literally means ‘self-eating’. It is a process in which a cell destroys old or defective cellular components, thereby releasing cellular building blocks including nucleotides, amino acids and fatty acids. Those degradation products can later be recycled by the cell to meet its metabolic needs. Autophagy is an essential intracellular process to ensure cell survival under stressful conditions (e.g. hypoxia, starvation and organelle damage). Different types of autophagy exist but, here, we will use the term to refer to macroautophagy. Autophagy is a complex multi-faceted process [63]. One putative biomarker is the level of LC3B-II, an essential protein during autophagosome formation and the level of scaffolding protein p62 [or sequestosome 1 (SQSTM1)] [64, 65]. Autophagy has both pro-tumour and anti-tumour functions, which may be both stage and tissue-type specific.

In early carcinogenesis, autophagy has a tumour suppressive role since it has an important quality control function and protects the cell by sequestering and eliminating defective cellular components, such as damaged mitochondria, and by maintaining cellular homeostasis [66, 67]. In addition, several autophagic proteins can directly suppress tumour formation (e.g. Beclin-1, UVRAG and Bif-1) and autophagy has been shown to degrade tumour promoting proteins as well (e.g. p62/SQSTM1) [68]. In line, deregulation of autophagy has been repeatedly associated with human cancers [67].

In contrast, autophagy can promote tumour growth in more advanced stages of cancer [69]. Pro-survival autophagy is induced in response to a variety of stressful conditions including but not limited to, starvation, loss of proteostasis, organelle damage and hypoxia. Some anti-cancer treatments can also induce pro-survival autophagy. Autophagic properties such as nutrient recycling can support cancer cell survival. Moreover, key regulators of cell growth can be degraded and the DNA damage response can be suppressed through increased autophagy [66–68]. Therefore, inhibition of autophagy can be an interesting anti-cancer strategy when cancer cells start depending on autophagy for survival, a moment called the autophagic switch [64, 70].

**Figure 1.**
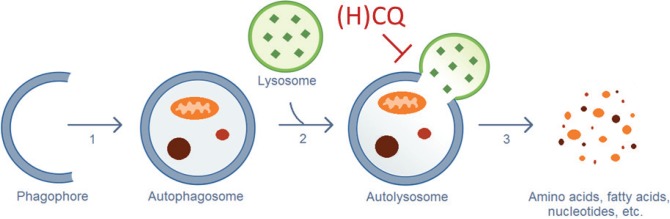
Autophagic process. (1) Elongation of the phagophore and vesicle formation. (2) Fusion of the autophagosome and a lysosome. (3) Destruction of the engulfed cellular components by lysosomal hydrolases. CQ and HCQ inhibit autophagy through interference with the lysosomal acidification (Step 2).

CQ and HCQ inhibit the autophagic flux at a late stage (Figure 1): the fusion of the autophagosomes with the lysosomes and subsequent degradation of the autolysosome. Upon entering the lysosomes, CQ and HQ become protonated, which leads to their entrapment in acidic lysosomes and an increase in the lysosomal pH, which inhibits the lysosomal degradative enzymes [71]. Loehberg *et al* [72] suggested that CQ might also modulate autophagy by modifying the PI3K/Akt/mTOR pathway.

In summary, autophagy plays a dual role in cancer and the success of autophagy inhibition, using the late stage inhibitors CQ and HCQ, depends on the timing and context. Autophagy is an interesting therapeutic target after the autophagic switch. However, the autophagy dependency of the tumour cells and any combinatory therapies can influence the sensitivity to autophagy inhibition, which will be discussed later.

#### Inhibition of the TLR9/nuclear factor kappa B signalling pathway

TLR9, a member of the Toll-like receptor family, is located in the endosomal compartment. This receptor recognises unmethylated single stranded DNA and is necessary for pathogen recognition and innate immune system activation. In cancer, expression and stimulation of TLR9 is linked with invasiveness, as shown in *in vitro* experiments [73–75]. Moreover, the expression levels of TLR9 are higher in hepatocellular carcinoma, oesophageal, lung, breast, gastric and prostate cancer cells as compared with adjacent noncancerous cells, and high expression is often linked with poor prognosis [73–76]. Because of this observation, it was suggested that TLR9 might be an appropriate anti-cancer target [73, 74, 76].

The TLR9-mediated activation of the NF-κB signalling pathway and the associated enhanced expression of matrix metalloproteinase-2 (MMP-2), MMP-7 and cyclo-oxygenase 2 mRNA, all factors associated with tumour progression and migration, can explain the role of TLR9 in cancer [73, 74]. At first, CQ was thought to inhibit this pathway by inhibiting endosomal acidification. However, CQ most likely modifies the structure of the nucleic acids responsible for TLR activation to prevent binding to TLRs [77]. An *in vitro* study also showed that invasion of brain cancer cells is hypoxia-induced through upregulation of TLR9 expression, which could be significantly inhibited by CQ [78].

In contrast, low expression of TLR9 is reported to be associated with a poorer prognosis in patients with triple-negative breast cancer. CQ had a promising effect on tumour growth and invasiveness, independent of the TLR9 status in triple-negative breast cancer cells *in vitro*, but it did not reduce the growth of orthotopic triple-negative breast cancer tumours *in vivo* [79, 80].

#### Inhibition of CXCL12/CXCR4 signalling

The interaction between the CXCR4 chemokine receptor and its ligand CXCL12 plays a major role in chemotaxis and adhesion of cells, and secretion of growth factors. In recent years, research has shown an association between CXCL12/CXCR4 signalling and cancer progression [81, 82]. This interaction is said to influence the invasive phenotype of pancreatic cancer for example.

In 2012, a CXCR4 small molecule antagonist (NSC56612), structurally resembling CQ and HCQ, was identified through *in silico* modelling of this receptor [82]. Next, CQ and HCQ were tested via *in vitro* assays, in which they were found to suppress pancreatic cancer cell proliferation [82, 83]. Mechanistic studies have shown that CQ, at least, partially inhibits CXCL12/CXCR4 signalling, as demonstrated via reduced phosphorylation of the extracellular signal-regulated kinase (ERK) and the signal transducer and activator of transcription 3 (STAT3). Interestingly, CQ and HCQ can induce CXCR4 internalisation in cancer stem cells, making these cells less sensitive to CXCL12 signals [83].

Furthermore, a study in a pancreatic cancer patient-derived xenograft model showed that CQ specifically targets highly aggressive cancer stem cells through inhibition of their self-renewal process. Thus, CQ could be useful to block cancer stem cell-metastasis and may be combined with other anti-cancer agents (e.g. gemcitabine) that target the bulk of the tumour [83].

#### Interference with the p53 pathway

The tumour suppressor protein p53 plays an essential role in maintaining an error-free genome and inducing cell death in case the damage is irretrievable. Therefore, it is a key protein in the prevention of tumour development [84].

Both *in vitro* and *in vivo* research has indicated that CQ can stabilise the p53 protein and activate the p53-dependent transcription of pro-apoptotic genes [17, 23, 24, 72, 84, 85]. Several hypotheses have been proposed to explain the underlying mechanism, but there is no definite answer yet. One of these hypotheses is that CQ intercalates in DNA, which leads to structural changes and thus induction of p53 [17, 85]. Moreover, the p53 activation by CQ might be mediated by the ataxia telangiectasia mutated protein, dependent on the cell type [17, 23, 24]

There is also some discussion about the relationship between the p53 status and the effects of autophagy inhibition on cancer development. Several studies report an accelerated tumour development when autophagy is inhibited in mice without p53 [35, 86, 87], but Yang *et al* [88] showed that that inhibition of autophagy could still have beneficial effect in p53 mutant tumours. Other studies confirmed that CQ exerts anti-cancer effects independent of the p53 pathway and the p53 status [88–90]. Synergy between the p53-dependent and -independent mechanisms of CQ is likely [17].

Recently, a p53-dependent mechanism was reported in which CQ induces tumour suppressor protein Par-4 secretion, triggering paracrine apoptosis of cancer cells and inhibition of tumour metastasis. This mechanism involves the CQ-dependent activation of p53 and the subsequent induction of Rab8b, which is necessary for transport of vesicles of Par-4 to the plasma membrane [91].

Moreover, CQ might prevent degradation of a p53-related protein, called Bcl homology-3-only protein p53 upregulated modulator of apoptosis (PUMA), as shown in mice studies. CQ increased the levels of PUMA, without affecting p53 in these studies [20, 92].

#### Other potential mechanisms of action

Additional mechanisms have been suggested, but they have not been studied to the same extent and will only be briefly described here.

In recent years, it has become clear that glutaminolysis plays an important role in metabolic processes associated with cancer cell proliferation and survival. Therefore, targeting glutaminolysis could provide novel approaches to improve cancer treatment [93]. It was shown that CQ affects glutamate dehydrogenase activity [94–96], which could be a potential mechanism of action in anti-cancer treatment. The hypothesis of inhibiting metabolic processes using metformin and CQ is currently being tested in one clinical trial with patients with isocitrate dehydrogenase 1 and 2 (IDH1/2)-mutated chondrosarcoma, glioma and intrahepatic cholangiocarcinoma [97].

CQ and HCQ can activate caspase-3 and modulate the Bcl-2/Bax ratio inducing apoptosis in CLL, B-cell CLL and glioblastoma cells [17, 89, 98–100]. CQ-mediated cell-cycle-arrest and apoptosis was observed in breast cancer cells and was associated with a decrease in protein levels/activity of polo-like kinase 1 (Plk-1), ERK1/2 Akt and cell division cycle 25C (CDC25C). The same study described induction of caspase-3-mediated spindle abnormalities and down regulation of the mitochondrial transmembrane potential by CQ [101]. A decreased lung cancer cell growth after low CQ concentrations was ascribed to an increased lysosomal volume and a phosphatidylcholine-specific phospholipase C involvement (PC-PLC). Higher CQ concentrations still induce apoptosis and necrosis, but likely via different processes [102].

Moreover, HCQ might affect acetylation status in the N-terminal lysines of histones H3 and H4, thus modulating cell growth and differentiation, as shown in human breast cancer cells [103].

In addition, CQ might directly affect Hedgehog signalling. Under normal conditions, this is a quiescent pathway, but activation can cause tumorigenesis and maintains cancer stem cells. Anti-cancer treatment options targeting this specific pathway have been explored, but this has yielded little results so far [104]. One study suggested that CQ might modulate protein levels of the Hedgehog signalling pathway (smoothened, patched and GLI1 proteins) [83].

CQ can inhibit hypoxia-stimulated metastasis via modulation of hypoxia-inducible factor 1α (HIF-1α), vascular endothelial growth factor (VEGF), and epithelial mesenchymal transition (EMT) as shown in a cholangiocarcinoma cell line [105].

In triple-negative breast cancer, CQ was shown to eliminate cancer stem cells through reduction of the expression of Janus-activated kinase 2 and DNA methyl transferase 1 [106] or through induction of mitochondrial dysfunction, subsequently causing oxidative DNA damage and impaired repair of double-stranded DNA breaks [107].

Of note, various studies showed growth inhibition of melanoma cells after CQ administration, but this inhibition was more pronounced in pigmented melanoma, which could be ascribed to CQ’s high affinity for melanin [108]. There is also some contradictory evidence about a potential link between Burkitt’s lymphoma incidence and CQ administration [109, 110].

### Modulation of tumour micro-environment

#### Immunomodulation

An increasing level of research is addressing the essential role of the immune system in cancer development. Activating the immune system against cancer cells is becoming a promising therapeutic approach [111], as immune cells have the ability to detect and destroy malignant cells [66].

Interestingly, autophagy and lysosomal function have been found to be involved in both innate and adaptive immunity [66]. Therefore, inhibitors of these processes such as CQ and HCQ could potentially modulate the immune system and subsequently influence tumour development. However, lysosomal function and autophagy have a dual role in the anti-tumour immune response. Activation of these processes could both activate and impair the immune response, dependent on the circumstances [66]. In addition, autophagy and lysosomal function affect the response of tumour cells to the immune system as well. For example, tumour cell autophagy can generate mediators that provoke an immune response via modulation of the tumour cell secretome and surface proteome, but it may also help tumour cells to escape the immune system [64, 66].

In general, the interaction between cancer cells and the immune system is complex and further research is warranted to determine when CQ or HCQ administration can lead to beneficial effects in the context of anti-tumour immunity [112]. This is particularly important if CQ or HCQ would be considered for use in combination with immunomodulation anti-cancer therapies.

#### Normalisation of the tumour vasculature

The tumour vasculature, responsible for supplying the tumour with nutrients and oxygen, is an important component in the tumour micro-environment and plays an essential role in tumour cell metastasis [113]. A first therapeutic approach is to destroy blood vessels in order to block the nutrient and oxygen supply to the tumour. However, accumulating evidence suggests that improving the highly abnormal tumour vessel structure, also called vessel normalisation, is preferred over anti-angiogenic therapy. The benefits of vessel normalisation include a decrease in tumour hypoxia, reduced cancer cell intravasation and metastasis, and an increase in chemotherapeutic drug delivery and response [114, 115].

A recent study showed that CQ normalises tumour vessels, independent of its autophagy inhibitory effect, through reduction of vessel density and improvement of cell alignment and formation of tight junctions. At the molecular level, CQ alters endosomal Notch1 trafficking and signalling in endothelial cells, hereby increasing the quiescent phenotype of the endothelial cells [26, 116]. Of note, systemic CQ administration has also been shown to reduce the vascular toxicity of the intratumorally administered, anti-tumour agent Transferrin-CRM107 in *in vivo* glioma models [117].

#### Disruption of the CAF—cancer cell interplay

The final interplay between the tumour and its microenvironment that may be influenced by CQ involves CAFs [118]. Glutamine and caveolin-1 are key players in this autophagy-mediated interplay, in which CAFs and tumour cells support each other through glutamine production/secretion and autophagy stimulation. Interestingly, this interplay can be uncoupled through the autophagy inhibitory or lysosomotropic activity of CQ, but the exact mechanism should still be clarified [119, 120].

### Synergism with approved anti-cancer drugs

Existing anti-cancer therapies often induce pro-survival autophagy in cancer cells, which is associated with therapeutic resistance. Because of their ability to inhibit autophagy, CQ and HCQ are able to sensitise tumour cells to chemotherapy and radiation. Therefore, these drugs are often tested in (pre)clinical research in combination with other anti-cancer therapies. Though, some caution is advised when concomitantly using these antimalarial drugs with other anti-cancer agents because autophagy can also be inhibited in normal cells, which causes unwanted toxicity (e.g. nephrotoxicity) [121].

CQ-mediated sensitisation to anti-cancer therapy has also been ascribed to autophagy-independent mechanisms. As mentioned earlier, chemotherapeutics can reach the tumour site more easily after tumour vessel normalisation [26, 116]. Moreover, CQ can prevent the entrapment of protonated chemotherapeutic drugs by buffering the extracellular tumour environment and intracellular acidic spaces [112]. For example, CQ can reduce the endosomal sequestration of certain drugs by raising the endosomal pH and, thus, increase their efficacy (e.g. doxorubicin, daunorubicin and mitoxantrone) [122–124]. Vezmar *et al* [125, 126] suggested that CQ influences multidrug resistance protein-mediated doxorubicin resistance by binding the multidrug resistance protein.

### Prediction of efficacy in individual patients

Autophagy dependency and metabolic stress levels of tumour cells vary widely depending on the tumour type and progression stage. Therefore, reliable measurements to predict tumour sensitivity to autophagy inhibition would be extremely useful for patient selection in clinical practice [67]. As mentioned earlier, the status of tumour suppressor p53 can affect CQ efficacy, but other CQ sensitivity indicators have been identified as well.

First, EGFR overexpressing tumour cells, high levels of STAT3 activity, loss of caveolin-1, Akt- and Myc- driven tumour cells, and argininosuccinate synthetase enzyme deficiency are all associated with a high autophagy dependency and are therefore more sensitive to CQ administration [16, 28, 119, 127–129]. Next, there is still discussion about the effect of the oncogenic BRAF (V600E) mutation on autophagy dependency of tumour cells [47, 130, 131]. In addition, evidence has shown that autophagy is induced by the tumour suppressor alternative reading frame, but it should still be clarified whether this is cytotoxic or protective autophagy before we can determine whether CQ administration would exert beneficial effects [132]. Moreover, oncogenic Ras, and especially Kras, mutation has also been suggested as an indicator of autophagy dependency and susceptibility to CQ [27, 133], but two other studies have reported that this mutation is not a reliable indicator [134, 135]. As mentioned earlier, however, HCQ has been shown to promote tumour growth in Ras-driven pancreatic tumours developing without p53 (Kras^G12D/+^ p53^–/–^) [35, 36]*.* Cells with the IDH1/2 mutations are metabolically vulnerable to CQ treatment, because they depend on glutaminolysis and autophagy, which is inhibited by CQ [97].

Autophagy dependency is higher in case of nutritional stress, as shown in mesothelioma cells [128], and neuroendocrine lung tumour cells are more sensitive to autophagy inhibition than non-neuroendocrine lung tumour cells [136].

Finally, an *in vitro* study in four human glioma cell lines observed that higher steady-state mitochondrial membrane potential values, representing mitochondrial stability, can predict cancer cell resistance to CQ treatment [137].

## Our take

The final goal of this literature review was to inform further research and trials on repurposing CQ and HCQ as anti-cancer agents, as done previously for other agents [138]. In addition, the ideal dose, route of administration, and therapeutic schedule that should be applied in anti-cancer therapy was explored. Finally, the potential difference in efficacy and toxicity between CQ and HCQ has been investigated.

### Efficacy of CQ and HCQ in anti-cancer therapy

The vast majority of preclinical studies on the effect of CQ monotherapy in cancer have reported a positive therapeutic effect, but the study parameters, doses, animal models and tumour types differ strongly between studies, complicating the interpretation of the results. Preclinical studies investigating the effect of HCQ in cancer are limited. Therefore, follow-up *in vivo* studies are warranted. A risk of publication bias exists so we cannot guarantee that all negative results have been reported.

Combination therapy with CQ or HCQ and existing anti-cancer therapies has been extensively studied in preclinical research, both *in vitro* and *in vivo*. The majority of these studies have reported an improved therapeutic efficacy as compared with monotherapy with existing anti-cancer drugs. Most studies hypothesise that CQ and HCQ could increase the efficacy of other anti-cancer drugs by blocking pro-survival autophagy. Because not all studies measured autophagy levels *in vivo,* it is difficult to determine to what extent the other proposed mechanisms play a role. Table 2 is limited to studies that tested CQ or HCQ in combination with conventional anti-cancer agents *in vivo*, but there are many other combinations that have only been tested *in vitro*.

Finally, multiple clinical trials have investigated, or are going to investigate, the use of CQ and HCQ in different cancer types, always in combination with other anti-cancer drugs. The availability of clinical results is limited now, as most trials are still recruiting or ongoing, and those that have been completed focused primarily on safety and tolerability of CQ and HCQ in cancer. In short, these drugs have been found safe and tolerable in all completed studies and the anti-cancer effect of both compounds is promising. However, as many clinical trials are still ongoing, a definite conclusion on the repurposing intent of CQ and HCQ in anti-cancer therapy is pending. Still, data from first clinical trials and additional preclinical data point to a potential positive implementation of these drugs in anti-cancer treatment.

### Doses, route of administration and therapeutic schedule

In preclinical experiments, varying CQ and HCQ doses have been used, but most of the applied doses can be extrapolated to human doses. However, whether the dose to achieve autophagy inhibition, induction of apoptosis and tumour normalisation is achievable in humans remains an open question that would require collecting additional data in humans [112]. Clinical trials have shown that daily doses between 150 and 500 mg for CQ and daily doses between 400 and 1200 mg for HCQ are safe and well tolerated, but two studies identified 600-mg HCQ daily as the MTD. HCQ is often administered twice daily to limit plasma fluctuations and toxicity. Of note, Pascolo recommended 10 mg/kg as the maximum realistic clinical dosage of CQ, but the recommended dose and MTD of CQ and HCQ might vary dependent on the tumour type and the concomitantly administered anti-cancer treatments.

Pascolo also suggests that timing of administration is of great importance. CQ must be administered after chemotherapy and not before, which is supported by data in a mouse model of colorectal cancer treated with gemcitabine [139].

### CQ or HCQ?

HCQ has been reported to have less side effects than CQ (e.g. less risk of retinal toxicity) [9, 140, 141], so it can be administered in higher doses for human use. Currently it is not clear yet whether there are differences in anti-cancer treatment efficacy between CQ and HCQ. The clinical trials that have already been completed suggest that CQ might be more efficacious than HCQ. However, no comparative clinical trial has been set up to confirm this hypothesis.

Yet, based on chemical structure, the altered safety and efficacy can be ascribed to the additional hydroxyl group in HCQ, causing pharmacokinetic differences that are essential for the working mechanism of the drugs (e.g. pKa alteration leading to differences in biprotonation and distribution) [14, 140].

### Next steps

More than 30 clinical trials are currently ongoing (Feb 2017). The results of these trials may indicate which tumour types are most sensitive to CQ and HCQ treatment, and which combination therapies can be beneficial. Additional preclinical studies could further characterise the most relevant mechanisms of action and their individual importance in anti-cancer therapy. Finally, CQ analogues and other more specific autophagy inhibitory agents are also under investigation for the treatment of cancer patients (e.g. Lys05) [142–146].

## Conclusion

CQ and HCQ have been studied in multiple preclinical cancer models and have demonstrated activity on several cancer-supporting pathways and in combination with a broad range of other therapies. Our review has highlighted the interesting multi-faceted actions of CQ and HCQ against cancer, making these drugs attractive for this complex disease [147, 148].

Even though it is too soon to make definite conclusions about the overall effect of CQ and HCQ in anti-cancer treatments, the clinical data already available are encouraging to further explore their potential as anti-cancer agents, with a preference for CQ. Until now, most clinical evidence was found in patients with glioblastoma and brain metastases and in patients with BRAF mutations, but some promising effects have been reported in patients with lung cancer, multiple myeloma and sarcoma as well. Although the side effects of CQ and HCQ are minor in comparison with conventional anti-cancer therapy, the possibility of retinal toxicity in trials planning long-term CQ and HCQ exposure requires the implementation of ophthalmologic monitoring. More than 30 clinical studies are currently evaluating HCQ and CQ in different cancers, most of them with the rationale to increase the efficacy of other anti-cancer therapies through inhibition of treatment-induced autophagy. The first clinical trials with CQ and HCQ have focused on the toxicity of different CQ doses in multiple populations and new trials should now focus on rigorous evaluation of efficacy.

## Conflicts of interest

The authors declare that they have no conflicts of interest.

## Author contributions

Primary authors: Ciska Verbaanderd and Gauthier Bouche. Contributing authors: Hannelore Maes, Marco Schaaf, Vikas P Sukhatme, Pan Pantziarka, Vidula Sukhatme and Patrizia Agostinis. All authors read and approved the final manuscript.

## Figures and Tables

**Table 1.** *In vivo* studies investigating the efficacy of CQ and HCQ monotherapy.

Reference	Animal model	Tumour type	Animal (H)CQ dose	HED[149]
Jutten *et al* [16]	NMRI-nu (nu/nu) female mice	Xenografts of U373-EGFRwt and U373 control cells	CQ: 60 mg/kg/day for seven consecutive days (IP)	292 mg/day
Kim *et al* [17]	NMRI nude mice	Xenografts of U87MG cells	CQ: Intracranial administration of 5μl with a concentration of 30 mM/day for 17 days	/
Song *et al* [18]	Male athymic BALB/c nu/nu mice	Xenografts of CD133+ and CD133- cells isolated from Huh 7 cells	CQ: 60 mg/kg, twice weekly (IP)	292 mg twice weekly
Hu *et al* [19]	Nude mice	Xenograft of HepG2-GFP human liver cancer cells	CQ: 80 mg/kg twice daily, on a 3-day-on/2-day-off schedule for 25 days (SC)	398 mg twice daily (3 day-on/2 day-off)
Lakhter *et al* [20]	NOD-SCID mice	Xenografts of SKMel23 cells	CQ: 25 mg/kg, twice weekly for 3 weeks (IP)	122 mg twice weekly
Zheng *et al* [21]	Female BALB/c mice	Transplantation of CT26 cells	CQ: 50 or 25 mg/kg/day for 28 days (IP)	243 or 122 mg/day
Jiang *et al* [22]	Female BALB/c mice	Transplantation of 4T1 mouse cells	CQ: 50 or 25 mg/kg/day for 28 days (IP)	243 or 122 mg/day
Loehberg *et al* [23]	Wistar-Furth virgin female rats	NMU-induced mammary adenocarcinoma (IP, 50 mg/kg)	CQ: 3.5 mg/kg/week for 3 weeks (IP)	34 mg/week
Loehberg *et al* [23]	BALB/c mice	Transplantation of mammary ducts from 7- to 8-week-old p53-null BALB/c mice	CQ: 3.5 mg/kg/week for 8 weeks (IP)	17 mg/week
Maclean *et al* [24]	ATM-null, p53-null mice (C57BL/6J) or Eμ-Myc transgenic mice (C57BL/6J)	Lymphoma	CQ: 3.5 mg/kg, every 5 days (combined oral/IP or IP alone)	17 mg every 5 days
Sun *et al* [25]	Male Sprague Dawley rats	DEN-induced hepatocarcinoma	CQ: 50 mg/kg, every 3 days during week 0 to 9 or during week 10 to 17 (IP)	486 mg every 3 days
Maes *et al* [26]	Immunocompetent syngeneic (C57/Bl6) or immunodeficient(nu/nu) mice	Xenografts of A375m and transplantation of B16-F10 mouse cells	CQ: 50 or 100 mg/kg/day (IP)	243 or 486 mg/day
Maycotte *et al* [28]	Female Nude nu/nu mice	Xenografts of MCF7 and MDAMB231 cells	CQ: 60 mg/kg/day (IP)	292 mg/day
Yang *et al* [27]	NCr nude mice (Taconic)	Xenografts of 8988T, H460 cells, and panc1 cells and an orthotopic PDAC model with 8988T cells grown in the pancreata	CQ: 60 mg/kg/day (IP)	292 mg/day
Hiraki *et al* [29]	Bashford cancer, Ehrlich ascites and solid cancer, MH134 tumour maintained in inbred strains Strong A, C3H, RIll, and RF mice, Yoshida ascites and solid tumours carried in Wistar and random-bred rats, and Brown-Pearce carcinoma transplanted in albino male rabbits		CQ: 6 - 15 mg/kg/day (IP, IV, SC, oral)	58 – 292 mg/day
Pellegrini *et al* [30]	Female NMRI nu/nu mice	Xenografts of HCT116 and HT29 cells	CQ: 20 mg/kg, every 2 days for 16 days (IP)	97 mg every 2 days
Dutta *et al* [32]	Female inbred F344 rats	Transplantation of R3230AC mammary adenocarcinoma	CQ: 45 mg/kg, 5 days a week for 25 days (IP)	438 mg 5 days a week
Yamaguchi *et al* [33]	Adult female C3H/HeN mice and adult male dd-mice	Transplantation of spontaneous C3H mammary carcinoma, Bashford carcinoma 63 and Ehrlich ascites tumours	CQ: 0.2 mg/2 days for 12 days (IP)	/
Chi *et al* [34]	Transgenic flies (Drosophila)	RasV12 tumours	CQ- containing medium (final concentration: 1 mg/ml)	/
Rosenfeldt *et al* [35]	KrasG12D/-p53-/- and KrasG12D/-p53+/+ mice	Pancreatic ductal adenocarcinoma	HCQ: 60 mg/kg/day (IP)	292 mg/day

Abbreviations: CQ (chloroquine), HCQ (hydroxychloroquine), EGFR (epidermal growth factor receptor), IP (intraperitoneal), NOD SCID mice (non-obese diabetic, severe combined immunodeficiency mice), NMU (N-methyl-N-nitrosourea), ATM (ataxia telangiectasia mutated), DEN (diethylnitrosamine), IV (intravenous), SC (subcutaneous), HED (Human Equivalent Dose).

**Table 2.** Overview of *in vivo* research combining known anti-cancer agents with either CQ or HCQ.

Reference	(H)CQ	Intervention	Animal model	Tumour type	Therapeutic effect of combination therapy
Golden *et al* [150]	CQ	Temozolomide (TMZ)	4- to 6-week-old male athymic nu/nu mice	U87MG glioma cells	Higher levels of the proapoptotic protein C/EBP homologous protein/growth arrest- and DNA damage-inducible gene 153 (CHOP/GADD-153)
Zanotto-Filho *et al* [151]	CQ	TMZ (+curcumin)	8-week-old male wistar rats	C6 brain cells	Autophagy inhibition and significantly reduced tumour growth
Gaudin *et al* [152]	CQ	Cyclophosphamide (Cytoxan)	Golden Syrian hamster	Melanoma and plasmacytoma	Sensitisation to cyclophosphamide
Lefort *et al* [153]	CQ	Cyclophosphamide (+Adriamycin)	6-week-old female Swiss nude mice	MDA-MB-231 human breast cancer cells	Significant tumour growth inhibition and reduction of lung metastases
Amaravadi *et al* [129]	CQ	Cyclophosphamide	8-to-10-week-old C57BL/6 ×129F1 mice	Myc/p53ERTAM lymphomas	Tumour growth inhibition and significant delay of tumour recurrence
Yu *et al* [154]	CQ	Cisplatin	4-to-6-week-old female BALB/c nu/nu mice	EC109/CDDP human oesophageal cells	Significantly lower tumour growth rate
Zhang *et al* [155]	CQ	Cisplatin	8-week-old female BALB/c mice	SGC7901 human gastric cancer cells	Significantly reduced tumour volume and weight
Zhao* et al* [156]	CQ	Cisplatin	5-to-6-week-old BALB/c nude mice	FaDu human hypopharyngeal cells	Prolonged survival
Ding *et al* [157]	CQ	Oxaliplatin	4-week-old male athymic BALB/c nude mice	Huh7 hepatocarcinoma cells	Significantly reduced tumour volume
Selvakumaran *et al* [158]	CQ	Oxaliplatin (+bevacizumab)	8-to-10-week-old female C.B.17 SCID mice	HT29 human colon carcinoma cells	Significant tumour growth delay
Liang *et al* [107]	CQ	Carboplatin	immunodeficient SCID-Beige mice	SUM159 cells breast cancer cells (orthotopic)	Significantly reduced tumour growth, decreased mitochondrial metabolic activity, decreased cell viability and increased levels of LC3b-II and p62
Balic *et al* [83]	CQ	Gemcitabine	Immuno-compromised mice	patient- derived PDAC tumour tissues	Effective tumour elimination and improved overall survival
Shoemaker *et al* [159]	CQ	5-FU	Young adult female C3H mice	C3HBA mammary carcinoma	Significantly reduced tumour size
Guo *et al* [160]	CQ	5-FU	5-week-old male athymic BALB/c nu/nu mice	SMMC-7721 hepatocarcinoma cells	Significantly reduced tumour volume and weight and significantly higher levels of apoptosis
Sasaki *et al* [161]	CQ	5-FU	6-week-old female BALB/c mice	Colon26 colon cancer cells	Significantly increased inhibition of tumour growth and increased number of apoptotic cells and proapoptotic protein expression levels
Shoemaker *et al* [162]	CQ	5-FU (+ 6-propyl-thiouracil)	Adult female C3H/He mice	C3HBA breast cancer cells	Significant tumour reduction
Xiong *et al* [163]	CQ	Daunorubicin	Female DBA/2 mice on a folate-deficient diet	L1210JF leukaemia cells	No effect
Arnold *et al* [164]	CQ	Etoposide	Female CBA/Ca mice	TLX5 murine ascitic tumour cells	Significant improvement in increased life span
Cook *et al* [165]	HCQ	Tamoxifen and faslodex	5-week-old, intact, athymic nude mice	Tamoxifen-resistant MCF7-RR and faslodex-resistant /Tamoxifen cross-resistant LCC9 ER+ breast cancer cells	Significantly reduced tumour size and tumour wet weight with HCQ and tamoxifen, no effect with faslodex and HCQ
Loehberg *et al* [72]	CQ	Everolimus	4-to-6-week-old, female NMRI nu/nu mice	MCF7 breast cancer cells	Significant tumour suppression
Seront *et al* [166]	CQ	Rapamycin	8-week–old female NMRI nude mice	MDA-MB-231 and MCF-7 breast cancer cells	Tumour growth reduction in mice implanted with large, hypoxic mammary tumours (not in smaller tumours)
Bray *et al* [167]	CQ	Temsirolimus	nude mice	RCC4 renal carcinoma cells	Significantly reduced tumour growth
Kaneko *et al* [168]	CQ	Temsirolimus	4-to-6-week-old BALB/c nu/nu and BALB/c mice	CaR-1, HT-29, colon26 colon cancer cells	Significantly reduced tumour growth
Xie *et al* [169]	HCQ	Temsirolimus	6-week-old male nude NCr Nu-M mice	UACC903 melanoma cells	Significantly tumour suppression and slower tumour growth
Rao *et al* [170]	CQ	Panobinostat	NOD/SCID mice	MB-231-luciferase mammary cells	Slight additional decrease in tumour growth as compared to CQ or Panobinostat monotherapy, but significant increase in survival time
Carew *et al* [171]	CQ	Vorinostat	Female nude BALB/c mice	HCT8 colon cancer cells	Significantly enhanced tumour reduction
Ding *et al* [172]	CQ	Bortezomib	6-to-8-week-old female BALB/c mice	HCT116 colon cancer cells	Significant inhibition of tumour growth and higher levels of apoptosis
Hui *et al* [173]	CQ	Bortezomib	nude mice	MHCC-97H and Huh-7 hepatocarcinoma tissues	Significantly reduced tumour growth and increased apoptosis
Tang *et al* [174]	CQ	Gefitinib	6-week-old male BALB/c nude mice	PC-9/wt and PC-9/gefB4 lung cancer cells	Significantly reduced tumour growth
Dragowska *et al* [175]	HCQ	Gefitinib	female Rag2M immune-compromised mice	JIMT-1 breast cancer cells	58% tumour reduction
Bokobza *et al* [176]	CQ	Gefitinib (+ Akt inhibitor)	BALB/c female nude mice	HCC-827 lung cancer cells	Significantly inhibited tumour growth compared to the control, addition of Akt inhibitor or chloroquine to gefitinib increased anti-tumour effects, but was not found to be significant.
Zou *et al* [177]	HCQ	Erlotinib	5-to-6-week-old athymic nude mice	H358 or H460 human NSCLC cells	Significant sensitisation to erlotinib therapy
Bellodi *et al* [178]	CQ	Imatinib	Sub-lethally irradiated C3H/HeJ mice	MigRI GFP-LC3b–transduced 32D-p210BCR/ABL cells	Significant sensitisation to imatinib therapy
Abdel-Aziz *et al* [179]	CQ	Sunitinib	Female Swiss albino mice	Ehrlich ascites carcinoma cells	Significantly reduced tumour growth and weight
Shimizu *et al* [180]	CQ	Sorafenib	BALB/c nude mice	Huh7 hepato-carcinoma cells	Significantly suppressed tumour growth
Shi *et al* [181]	CQ	Sorafenib	Male athymic BALB/c nude mice	MHCC97-L hepatocellular cells	Significantly reduced tumour growth and increased apoptosis
Ji *et al* [182]	CQ	Crizotinib	6- to 7-week-old female CD-1 nude mice	crizotinib-resistant H3122CR-1 lung cancer cells	Sensitisation of drug resistant lung cancer cells to crizotinib
You *et al* [183]	HCQ	Crizotinib	5-to-6-week-old female athymic BALB/c nude mice	SPC-A1 human lung cancer cells	Significantly reduced tumour growth and increased apoptosis
Mitou *et al* [184]	CQ	Crizotinib	6-week-old female NOD-SCID mice	Karpas-299 lymphoma cells	Significantly reduced tumour growth and increased apoptosis
Shen *et al* [185]	CQ	Vandetanib	6-to-8-week-old female BALB/c nude mice	U251 glioblastoma cells	Significantly reduced tumour growth and increased apoptosis
Hu *et al* [186]	CQ	Bevacizumab	6-to-8-week-old female BALB/c nu/nu mice	1) GBM39 primary glioma cells 2) Subcutaneous U87MG glioma cells 3) G55 glioma cells 4) patient-specimen derived SF8244 cells	Significantly suppressed GBM39, U87MG, G55, and patient specimen-derived SF8244 tumour growth
Selvakumaran *et al* [158]	CQ	Bevacizumab (+ oxaliplatin)	8-to-10-week-old female C.B.17 SCID mice	HT29 human colon carcinoma cells	Significantly delayed tumour growth
Cufi *et al* [187]	CQ	Trastuzumab	4-to-5-week-old female athymic nude mice	JIMT-1 breast cancer cells	Significantly reduced tumour growth and increased Bax/Bcl-2 ratio
Gaudin *et al* [152]	CQ	Radiotherapy	Golden Syrian hamster	Melanoma and plasma cytoma	Increased sensitivity of melanoma and plasma cytoma tumour cells to X-rays
Ratikan *et al* [188]	CQ	Radiotherapy	6-week-old female H-2 3H/Sed//Kam and H-2 Rag2-/-, gamma c -/-mice	MCaK breast cancer cells	Significantly higher cure rate, delayed tumour growth and enhanced immunogenicity
Wei *et al* [189]	CQ	PDT	NOD/SCID mice	PROM1/CD133+ colorectal cancer stem cells	Restoration of sensitivity to PDT
Liang *et al* [190]	CQ	HDIL-2	8-to-10-week-old female C57BL/6 (B6, H-2b) mice	luciferase-labeled mouse MC38 colorectal cancer cells	Significantly reduced tumour growth and prolonged survival time
Thomas *et al* [191]	CQ	Nelfinavir Celecoxib	Athymic mice	MDA-MB-468 and MCF-7 breast cancer cells	Triple-drug treatment displayed obvious anti-cancer effects in both TNBC (MDA-MB-468) and non-TNBC (MCF-7) xenograft (=proof of principle study, more extensive *in vivo* experiments needed)
Harhaji-Trajkovic *et al* [192]	CQ	Caloric restriction	5-to-6-week-old female C57BL/6 mice	B16 melanoma cells	Combination of CQ and caloric restriction almost completely abolished B16 melanoma growth
Thomas *et al* [193]	CQ	Hyperthermia	male white Ajax mice	C-1300 murine neuroblastoma	! Increased tumour growth and metastasis
Gao *et al* [194]	CQ	TACE	Adult New Zealand White rabbits	VX2 liver tumours	Significantly reduced tumour volume and growth rate

Abbreviations: CQ (chloroquine), HCQ (hydroxychloroquine), SCID (severe combined immunodeficiency mice), PDAC (pancreatic ductal adenocarcinoma), 5-FU (5-fluorouracil) NOD (non-obese diabetic), wt (wild-type), PDT (photodynamic therapy), HDIL-2 (high-dose interleukin-2), TACE (transcatheter arterial chemoembolisation)

**Table 3.** Information on clinical trials investigating CQ use in cancer (Source: ClinicalTrials.gov).

ClinicalTrials.gov ID	Type of cancer	Intervention	Study Phase	Location	Status	First received	Last verified
NCT00224978	GBM	CQ (+ conventional treatment)	Phase 3	Mexico	Completed	Sept 2005	Nov 2009
NCT01438177	Multiple myeloma	CQ, Velcade, Cyclophosphamide	Phase 2	US	Completed, has results	Sept 2011	June 2016
NCT01727531	Brain metastasis	CQ, Radiation therapy	Not provided	US	Completed	Nov 2012	Apr 2015
NCT01777477	Pancreatic cancer	CQ, Gemcitabine	Phase 1	Switzerland	Completed	Jan 2013	Sept 2015
NCT01894633	Brain metastasis	CQ, Radiotherapy	Phase 2	Mexico	Terminated	June 2013	July 2013
NCT01469455	Local metastatic melanoma	CQ, DT01, Radiotherapy	Phase 1	France	Completed	Oct 2011	June 2016
NCT01023477	Ductal carcinoma in situ	CQ (Procedure: breast biopsy)	Phase 1 - 2	US	Ongoing	Dec 2009	Sept 2016
NCT00969306	Small cell lung cancer	CQ	Phase 1	The Netherlands	Recruiting	Aug 2009	Feb 2016
NCT01446016	Breast cancer	CQ, Taxane, Taxotere, Abraxane, Ixabepilone	Phase 2	US	Recruiting	Sept 2011	Sept 2016
NCT01575782	Small cell lung cancer	CQ, Radiotherapy	Phase 1	The Netherlands	Recruiting	Apr 2012	Sept 2016
NCT02071537	Advanced solid tumours	CQ, Carboplatin, Gemcitabine	Phase 1	US	Recruiting	Feb 2014	Dec 2015
NCT02333890	Breast cancer	CQ (and placebo) (prior to surgery)	Phase 2	Canada	Recruiting	Jan 2015	Nov 2016
NCT02366884	Neoplasms	Anti-Bacterial Agents, Anti-Fungal Agents, Anti-Protozoal Agents	Phase 2	Mexico	Recruiting	Feb 2015	Aug 2015
NCT02496741	Glioma, Cholangiocarcinoma, Chondrosarcoma	CQ, Metformin	Phase 1 - 2	The Netherlands	Recruiting	June 2015	Nov 2015
NCT02378532	GBM	CQ, Radiotherapy, Temozolomide	Phase 1	The Netherlands	Recruiting	Feb 2015	Aug 2016
NCT02432417	Glioblastoma, Astrocytoma (Grade IV)	CQ, Radiotherapy	Phase 2	Not provided	Not yet recruiting	Apr 2015	Apr 2016
NCT03243461	Glioblastoma WHO Grade IV, Diffuse Mid-line Glioma Histone 3 K27M, WHO Grade IV Anaplastic Astrocytoma WHO Grade III, Diffuse Intrinsic Pontine Glioma, Gliomatosis Cerebri	Radiochemotherapy with Temozolomide, Valproic Acid or Chloroquine	Phase 3	Germany	Not yet recruiting	Aug 2017	Oct 2017

**Table 4.** Information on clinical trials investigating HCQ use in cancer (Source: ClinicalTrials.gov).

ClinicalTrials.gov ID	Type of cancer	Intervention	Study Phase	Location	Status	First received	Last verified
NCT00765765	Breast cancer	HCQ, Ixabepilone	Phase 1 - 2	US	Terminated, has results	Oct 2008	Nov 2013
NCT00786682	Prostate cancer	HCQ, Docetaxel	Phase 2	US	Terminated, has results	Nov 2008	Sept 2013
NCT00728845	Lung cancer	HCQ, Bevacizumab, Carboplatin, Paclitaxel	Phase 1 - 2	US	Terminated, has results	Aug 2008	Sept 2013
NCT01026844	Non-small cell lung cancer	HCQ, Erlotinib	Phase 1	US	Terminated, has results	Dec 2009	June 2013
NCT01842594	Soft tissue sarcoma	HCQ, Sirolimus	Phase 2	Taiwan	Terminated, has results	Dec 2012	Oct 2015
NCT01144169	Renal cell carcinoma	HCQ (prior to surgery)	Phase 1	US	Terminated	June 2010	Oct 2016
NCT01417403	Bone metastases unspecified adult solid tumour	HCQ, Radiation therapy	Phase 1	US	Terminated	Aug 2011	Feb 2015
NCT00771056	B-cell chronic lymphocytic leukaemia	HCQ	Phase 2	US	Terminated	Oct 2008	Aug 2016
NCT00714181	Unspecified adult solid tumour	HCQ, Temozolomide	Phase 1	US	Completed	July 2008	Feb 2016
NCT01396200	Multiple myeloma	HCQ, Rapamycin, Cyclophosphamide, Dexamethasone	Phase 0	US	Completed	July 2011	Feb 2013
NCT01634893	Refractory or relapsed solid tumours	HCQ, Sorafenib	Phase 1	US	Completed	July 2012	Mar 2016
NCT01828476	Prostate cancer	HCQ, Abiraterone, ABT-263	Phase 2	US	Completed	Mar 2013	Mar 2016
NCT01006369	Colorectal cancer	HCQ, Bevacizumab, FOLFOX6, XELOX regimen (capecitabine, oxaliplatin)	Phase 2	US	Suspended	Oct 2009	Dec 2014
NCT00726596	Prostate cancer	HCQ	Phase 2	US	Ongoing	July 2008	Dec 2015
NCT00813423	Adult solid neoplasm	HCQ, Sunitinib malate	Phase 1	US	Ongoing	Dec 2008	Nov 2016
NCT00909831	Unspecified adult solid tumour	HCQ, Temsirolimus	Phase 1	US	Ongoing	May 2009	Feb 2016
NCT00962845	Melanoma	HCQ (prior to surgery)	Phase 0	US	Ongoing	Aug 2009	July 2016
NCT00977470	Non-small cell lung cancer	HCQ, Erlotinib	Phase 2	US	Ongoing	Sept 2009	Sept 2016
NCT01128296	Pancreatic cancer	HCQ, Gemcitabine (prior to surgery)	Phase 1 - 2	US	Ongoing	May 2010	Jan 2015
NCT01273805	Pancreatic cancer	HCQ	Phase 2	US	Ongoing	Jan 2011	Jan 2016
NCT01480154	Advanced solid tumours, melanoma, prostate or kidney cancer	HCQ, Akt Inhibitor MK2206	Phase 1	US	Ongoing	Nov 2011	Feb 2016
NCT01689987	Relapsed or refractory multiple myeloma	HCQ, Cyclophosphamide, Dexamethasone, Sirolimus	Phase 1	US	Ongoing	Sept 2012	Aug 2016
NCT01897116	Melanoma	HCQ, Vemurafenib	Phase 1	US	Ongoing	June 2013	July 2016
NCT02421575	Prostate cancer	HCQ (before prostatectomy or local therapy)	Phase 0	US	Ongoing	Dec 2014	July 2016
NCT01494155	Pancreatic cancer	HCQ, Capecitabine, Radiation: Proton or Photon Radiation Therapy	Phase 2	US	Ongoing	July 2011	Sept 2016
NCT01602588	Glioblastoma	HCQ, Short Course radiotherapy	Phase 2	UK	Ongoing	May 2012	Nov 2016
NCT02470468	Stage IV non-small cell lung cancer	DCVAC, Standard of Care Chemotherapy (Carboplatin, Paclitaxel), Immune enhancers (Interferon-α and HCQ)	Phase 1 - 2	Czech Republic and Slovakia	Ongoing	June 2015	Nov 2016
NCT01023737	Malignant solid tumour	HCQ, Vorinostat	Phase 1	US	Recruiting	July 2009	Sept 2016
NCT01206530	Colorectal cancer	HCQ, Oxaliplatin, Leucovorin, 5-fluorouracil, Bevacizumab	Phase 1 - 2	US	Recruiting	Sept 2010	Sept 2016
NCT01266057	Advanced cancers	HCQ, Sirolimus, Vorinostat	Phase 1	US	Recruiting	Dec 2010	Nov 2016
NCT01510119	Renal cell carcinoma	HCQ, RAD001	Phase 1 - 2	US	Recruiting	Jan 2012	Dec 2015
NCT01506973	Advanced and metastatic adenocarcinoma	HCQ, Gemcitabine/abraxane	Phase 1 - 2	US	Recruiting	Jan 2012	Sept 2016
NCT01550367	Metastatic renal cell carcinoma	HCQ, IL-2	Phase 1 - 2	US	Recruiting	Feb 2012	May 2015
NCT01649947	Non-small cell lung cancer	HCQ, Paclitaxel, Carboplatin, Bevacizumab	Phase 2	US	Recruiting	July 2012	July 2016
NCT01978184	Pancreatic cancer	HCQ, Gemcitabine, Abraxane	Phase 2	US	Recruiting	Oct 2013	Dec 2015
NCT02013778	Hepatocellular carcinoma	HCQ, TACE	Phase 1 - 2	US	Recruiting	Dec 2013	Sept 2016
NCT02232243	Solid tumour	HCQ (prior to surgery)	Phase 1	US	Recruiting	Sept 2014	Oct 2016
NCT02257424	Advanced BRAF mutant melanoma	HCQ, Dabrafenib, Trametinib	Phase 1 - 2	US	Recruiting	Oct 2014	June 2016
NCT02316340	Colorectal cancer	HCQ, Vorinostat, Regorafenib	Phase 2	US	Recruiting	Dec 2014	Sept 2016
NCT02414776	Oestrogen receptor positive breast cancer	HCQ, Hormonal therapy	Phase 1 (1b/2)	US	Recruiting	Jan 2015	Apr 2015
NCT02631252	Acute myeloid leukaemia	HCQ, Mitoxantrone, Etoposide	Phase 1	US	Not yet recruiting	Dec 2015	Dec 2015
NCT02722369	Small cell lung cancer	HCQ, Gemcitabine, Carboplatin, Etoposide	Phase 2	Not provided	Not yet recruiting	Mar 2016	Nov 2016
NCT00486603	Brain and central nervous system tumours	HCQ, Temozolomide, Radiation	Phase 1 - 2	US	Unknown	June 2007	May 2012
NCT00568880	Multiple myeloma and plasma cell neoplasms	HCQ, Bortezomib	Phase 3	US	Unknown	Dec 2007	July 2009
NCT00809237	Non-small cell lung cancer	HCQ, Gefitinib	Phase 1 - 2	Singapore	Unknown	Dec 2008	Dec 2013
NCT01227135	Chronic myeloid leukaemia	HCQ, Imatinibmesylate	Phase 2	UK	Unknown	Oct 2010	Nov 2011
NCT01292408	Breast cancer	HCQ	Phase 2	The Netherlands	Unknown	Dec 2010	Jan 2012

Abbreviations: FOLFOX6 (folinic acid – 5- fluorouracil – oxaliplatin), XELOX (capecitabine – oxaliplatin), IL-2 (interleukin-2), TACE (trans catheter arterial chemoembolisation), DCVAC (dendritic-cell based immunotherapy)

**Table 5.** Publications reporting clinical trial results on CQ use in cancer.

Article	Tumour type	Phase	Intervention	CQ dose	# patients	Therapeutic response
Briceño *et al* [38]	Glioblastoma multiforme	Unknown	CQ + conventional cancer treatment	150 mg/day	18 (9 CQ + 9 control)	Positive
Sotelo *et al* [39]	Glioblastoma multiforme	Phase 3	CQ + conventional cancer treatment	150 mg/day	30 (15 CQ + 15 control)	Partial
Briceño *et al* [41]	Glioblastoma multiforme	Retrospective study based on patient data	CQ + conventional cancer treatment	150 mg/day	123 (41 CQ + 82 control)	Positive
Rojas-Puentes *et al* [43]	Brain metastases	Phase 2	CQ + radiotherapy	150 mg/day	73 (39 CQ + 34 control)	Partial
Eldredge *et al* [44]	Brain metastases	Unknown	CQ + radiotherapy	250 mg/day	20 (all CQ, no control)	Partial
Montanari *et al* [49]	Relapsed and refractory multiple myeloma	Phase 1 - 2	CQ + bortezomib + cyclophosphamide	500 mg/day	8 (all CQ, no control)	Partial
Kyle *et al* [50]	Multiple myeloma	Unknown	CQ + prednisone + cyclophosphamide + caffeine	2x 250 mg/day	38 (18 CQ + 20 control)	Absent

**Table 6.** Publications reporting clinical trial results on HCQ use in cancer.

Article	Tumour type	Phase	Intervention	HCQ dose	# patients	Therapeutic response
Rangwala *et al* [51]	Advanced solid tumours and melanoma	Phase 1	HCQ + temsirolimus	RD: 2x 600 mg/day	39 (all HCQ, no control)	Partial
Rangwala *et al* [52]	Advanced solid tumours and melanoma	Phase 1	HCQ + temozolomide	RD: 2x 600 mg/day	40 (all HCQ, no control)	Partial
Mahalingam *et al* [53]	Advanced solid tumours	Phase 1	HCQ + vorinostat	MTD: 600 mg/day	27 (all HCQ, no control)	Partial
Chi *et al* [54]	Stage IV solid tumours	Pilot	HCQ + sirolimus + chemotherapy	400 mg/day	25 (all HCQ, no control)	Partial
Rosenfeld *et al* [55]	GBM	Phase 1–2	HCQ + radiotherapy + temozolomide	MTD: 600 mg/day	92 (all HCQ, no control)	Absent
Goldberg *et al* [57]	Advanced NSCLC	Phase 1	HCQ + erlotinib	RD: 1000 mg/day	27 (all HCQ, no control)	Partial
Vogl *et al* [59]	Relapsed and refractory multiple myeloma	Phase 1	HCQ + bortezomib	RD: 2x 600 mg/day	25 (all HCQ, no control)	Partial
Boone *et al* [60]	Pancreatic adenocarcinoma	Phase 1–2	HCQ + gemcitabine	RD: 1200 mg/day	35 (all HCQ, no control)	Partial
Wolpin *et al* [61]	Metastatic pancreatic adenocarcinoma	Phase 2	HCQ	400 and 600 mg/day	20 (all HCQ, no control)	Absent
Chi *et al* [62]	Sarcoma	Phase 2	HCQ + sirolimus	2x 200 mg/day	10 (all HCQ, no control)	Absent, study was closed prematurely

Abbreviations: MTD (maximal tolerated dose), RD (recommended dose), NSCLC (non-small cell lung cancer)
